# Hypoinsulinaemic, hypoketotic hypoglycaemia due to mosaic genetic activation of PI3-kinase

**DOI:** 10.1530/EJE-17-0132

**Published:** 2017-05-30

**Authors:** Sarah M Leiter, Victoria E R Parker, Alena Welters, Rachel Knox, Nuno Rocha, Graeme Clark, Felicity Payne, Luca Lotta, Julie Harris, Julio Guerrero-Fernández, Isabel González-Casado, Sixto García-Miñaur, Gema Gordo, Nick Wareham, Víctor Martínez-Glez, Michael Allison, Stephen O’Rahilly, Inês Barroso, Thomas Meissner, Susan Davies, Khalid Hussain, Karen Temple, Ana-Coral Barreda-Bonis, Sebastian Kummer, Robert K Semple

**Affiliations:** 1 Metabolic Research LaboratoriesWellcome Trust-MRC Institute of Metabolic Science, University of Cambridge, Cambridge, UK; 2 The National Institute for Health ResearchCambridge Biomedical Research Centre, Cambridge, UK; 3 Department of General PaediatricsNeonatology and Paediatric Cardiology, University Children’s Hospital, Düsseldorf, Germany; 4 Department of Molecular GeneticsAddenbrooke’s Hospital, Cambridge, UK; 5 Wellcome Trust Sanger InstituteHinxton, Cambridge, UK; 6 MRC Epidemiology UnitUniversity of Cambridge, Cambridge, UK; 7 Departments of Paediatric Endocrinology; 8 Departments of Clinical and Molecular GeneticsLa Paz Hospital, Madrid, Spain; 9 Departments of Hepatology; 10 Departments of HistopathologyAddenbrooke’s Hospital, Cambridge, UK; 11 Institute of Child HealthUniversity College London, London, UK; 12 Department of Clinical GeneticsUniversity Hospital Southampton, Southampton, UK

## Abstract

**Objective:**

Genetic activation of the insulin signal-transducing kinase *AKT2* causes syndromic hypoketotic hypoglycaemia without elevated insulin. Mosaic activating mutations in class 1A phospatidylinositol-3-kinase (PI3K), upstream from AKT2 in insulin signalling, are known to cause segmental overgrowth, but the metabolic consequences have not been systematically reported. We assess the metabolic phenotype of 22 patients with mosaic activating mutations affecting PI3K, thereby providing new insight into the metabolic function of this complex node in insulin signal transduction.

**Methods:**

Three patients with megalencephaly, diffuse asymmetric overgrowth, hypoketotic, hypoinsulinaemic hypoglycaemia and no *AKT2* mutation underwent further genetic, clinical and metabolic investigation. Signalling in dermal fibroblasts from one patient and efficacy of the mTOR inhibitor Sirolimus on pathway activation were examined. Finally, the metabolic profile of a cohort of 19 further patients with mosaic activating mutations in PI3K was assessed.

**Results:**

In the first three patients, mosaic mutations in *PIK3CA* (p.Gly118Asp or p.Glu726Lys) or *PIK3R2* (p.Gly373Arg) were found. In different tissue samples available from one patient, the *PIK3CA* p.Glu726Lys mutation was present at burdens from 24% to 42%, with the highest level in the liver. Dermal fibroblasts showed increased basal AKT phosphorylation which was potently suppressed by Sirolimus. Nineteen further patients with mosaic mutations in *PIK3CA* had neither clinical nor biochemical evidence of hypoglycaemia.

**Conclusions:**

Mosaic mutations activating class 1A PI3K cause severe non-ketotic hypoglycaemia in a subset of patients, with the metabolic phenotype presumably related to the extent of mosaicism within the liver. mTOR or PI3K inhibitors offer the prospect for future therapy.

## Introduction

Transient neonatal hypoglycaemia is common, often precipitated by inadequate deposition of energy stores *in utero* and/or perinatal stress. In contrast, persisting hypoglycaemia is often caused by a genetic disorder, and may be insulin dependent or insulin independent (1, 2, 3). The former is usually caused by congenital hyperinsulinism (CHI), or occasionally extreme insulin resistance (4). CHI-related hypoglycaemia features suppressed plasma ketones and free fatty acids but detectable plasma insulin, while glucagon stimulation characteristically increases blood glucose by greater than 30 mg/dL (5). Carbohydrate requirement to maintain euglycaemia in CHI is high, with intravenous glucose infusion rates usually exceeding 8 mg/kg/min in neonates and infants (2). Non-insulin-dependent hypoglycaemia may be caused by inherited metabolic diseases including glycogen storage or fatty acid oxidation disorders (6, 7).

**Figure 1:**
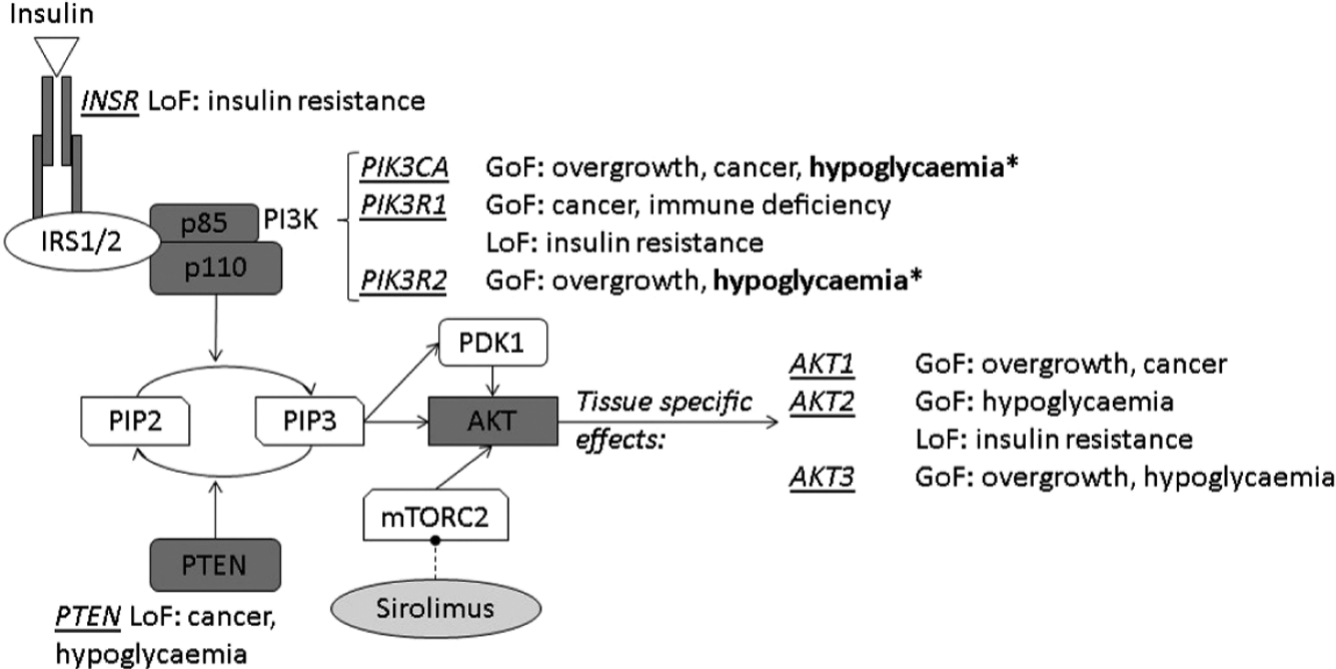
Schematic overview of INSR/PI3K/AKT signalling, showing known monogenic disorders. Asterisks (*) denote mutations described in this report. GoF, gain of function; LoF, loss of function.

We previously described a syndromic form of hypoglycaemia whose metabolic profile resembles CHI, yet in which neither insulin nor insulin-like molecules can be detected during hypoglycaemia (8). It is caused by the p.Glu17Lys mutation in the kinase *AKT2*, a critical mediator of insulin action (9). This mutation partly uncouples AKT2 activation from insulin-stimulated phosphatidylinositol-3-kinase (PI3K) activity by permitting binding of AKT2 to the PI3K substrate PIP_2_ in addition to its product phosphatidylinositol (3,4,5)-trisphosphate (PIP_3_) (Fig. 1) (10). This results in low-level cellular responses mimicking the presence of insulin.

Activating mutations in other components of the PI3K/AKT pathway are common in cancer (11, 12), and in isolation, in mosaic form, cause segmental overgrowth (13, 14, 15). The most commonly mutated gene is *PIK3CA*, encoding the p110α catalytic subunit of PI3K (13). Mutations in other genes including *PIK3R2*, encoding the p85β regulatory subunit of PI3K, are also described (16). *PIK3CA*-associated overgrowth in mosaic syndromes is known to affect venous and lymphatic vessels, adipose tissue, bone, muscle and neural tissue. Notably, the timing and site of the mutation gives rise to different patterns within the body, ranging from very specific localised/focal forms to diffuse manifestations involving multiple tissues. Additionally, the strength of genetic activation may further determine the severity of tissue-specific symptoms, resulting in pronounced phenotypic variability of these disorders, potentially also affecting metabolic and/or endocrine tissues. *PIK3R2* mutations lead to Megalencephaly–Polymicrogyria–Polydactyly–Hydrocephalus (MPPH) syndrome, which is predominantly characterised by brain overgrowth and neurological abnormalities; mutations are often germline rather than mosaic. To date, although PIK3CA has been proven in numerous genetic and pharmacological studies to be critical for the metabolic effects exerted by insulin, and despite scattered mentions of hypoglycaemia in MCAP (17, 18), the metabolic phenotype has not been examined in detail.

We now describe three patients with early-onset, severe, non-ketotic hypoglycaemia associated with segmental overgrowth and activating mutations in *PIK3CA* or *PIK3R2*, and assess the PI3K pathway dysregulation *in vitro* in patients’ fibroblasts. Furthermore, we systematically survey the metabolic profile of a cohort of patients with mosaic PI3K activation ascertained through segmental overgrowth.

## Subjects and methods

### Cohort studied and ethical approval

Informed consent was obtained from all participants, research was approved by relevant research ethics committees, and the study was performed in accordance with the Declaration of Helsinki. For the cohort analysis, all patients with mosaic activating *PIK3CA* mutations from a study of segmental overgrowth for whom metabolic data were available were also assessed, encompassing volunteers with diagnoses of CLOVES (Congenital lipomatous overgrowth with vascular, epidermal, and skeletal anomalies) syndrome (OMIM #612918) (19), Klippel–Trenaunay (KT) syndrome (OMIM #149000) (20), Fibroadipose hyperplasia (13), macrodactyly or primary muscle overgrowth (21) or Megalencephaly–Capillary Malformation (MCAP) (OMIM #602501) (22). Biochemical evaluations were performed in accredited diagnostic laboratories.

### Genetic studies

For Sanger sequencing, exons and flanking regions were PCR amplified before sequencing using ABI’s BigDye Terminator Mix, purification using AgenCount AMPure Beads, capillary electrophoresis and analysis using Sequencher software (GeneCodes). Exome-wide sequencing for P1 and parents was performed and analysed as previously described (23). *PIK3CA* p.Glu726Lys mutation burden was determined by custom-designed fluorescence-based restriction fragment assay (as described in Supplementary Online Material, see section on supplementary data given at the end of this article). P3 salivary DNA was sequenced using a custom panel of overgrowth-related genes on an Illumina MiSeq platform with preceding target enrichment. Further method and details are described in the Supplementary Online Material.

### Cellular studies

Dermal fibroblasts were cultured from punch biopsies and maintained in DMEM supplemented with 10% Foetal Bovine Serum (FBS) containing 100 U/mL Penicillin, 100 µg/mL Streptomycin and 4 mM l-Glutamine (all Sigma). For serum starvation, FBS was substituted by 0.5% Bovine serum albumin (Sigma). For signalling studies, fibroblasts were grown to confluence and washed twice with PBS before serum starvation for 24 h. Sirolimus (Sigma) was diluted in DMSO to 10 μM. Cells were pre-treated with Sirolimus or DMSO for 48 h prior to continued treatment during serum starvation. Cells were frozen in liquid nitrogen and stored at −80°C until processing.

AKT phosphorylation was determined using ELISAs for pThr308/309 and pSer473/474 (eBiosciences #85-86044 & #85-86042) according to manufacturer’s instructions. Protein quantification was performed by DC Protein assay (BioRad). For immunoblotting, lysates were resolved by electrophoresis on 8% Bis-Tris gels (NuPage, Thermo Fisher) before transfer onto nitrocellulose membranes using the iBlot system (Life Technologies). Membranes were blocked in 4% BSA/TBST prior to primary antibody exposure for 16 h. Following the addition of secondary antibodies, blots were imaged on a BioRad ChemiDoc. The following antibodies were used: anti-calnexin (AbCam #22595), anti-AKT(1/2/3) (CST #9272) and anti-rabbit HRP conjugate (CST #7074). ImageLab software (BioRad) was used to determine band intensity.

### Statistical analysis

Statistical analysis was undertaken using GraphPad Prism v.6. Log transformation was performed prior to analysis as the D’Agostino–Pearson test showed non-normal distribution of results. One-way analysis of variance (ANOVA) was undertaken followed by Dunnett’s test for comparison of patient against control cells.

## Results

### Clinical histories

**Figure 2:**
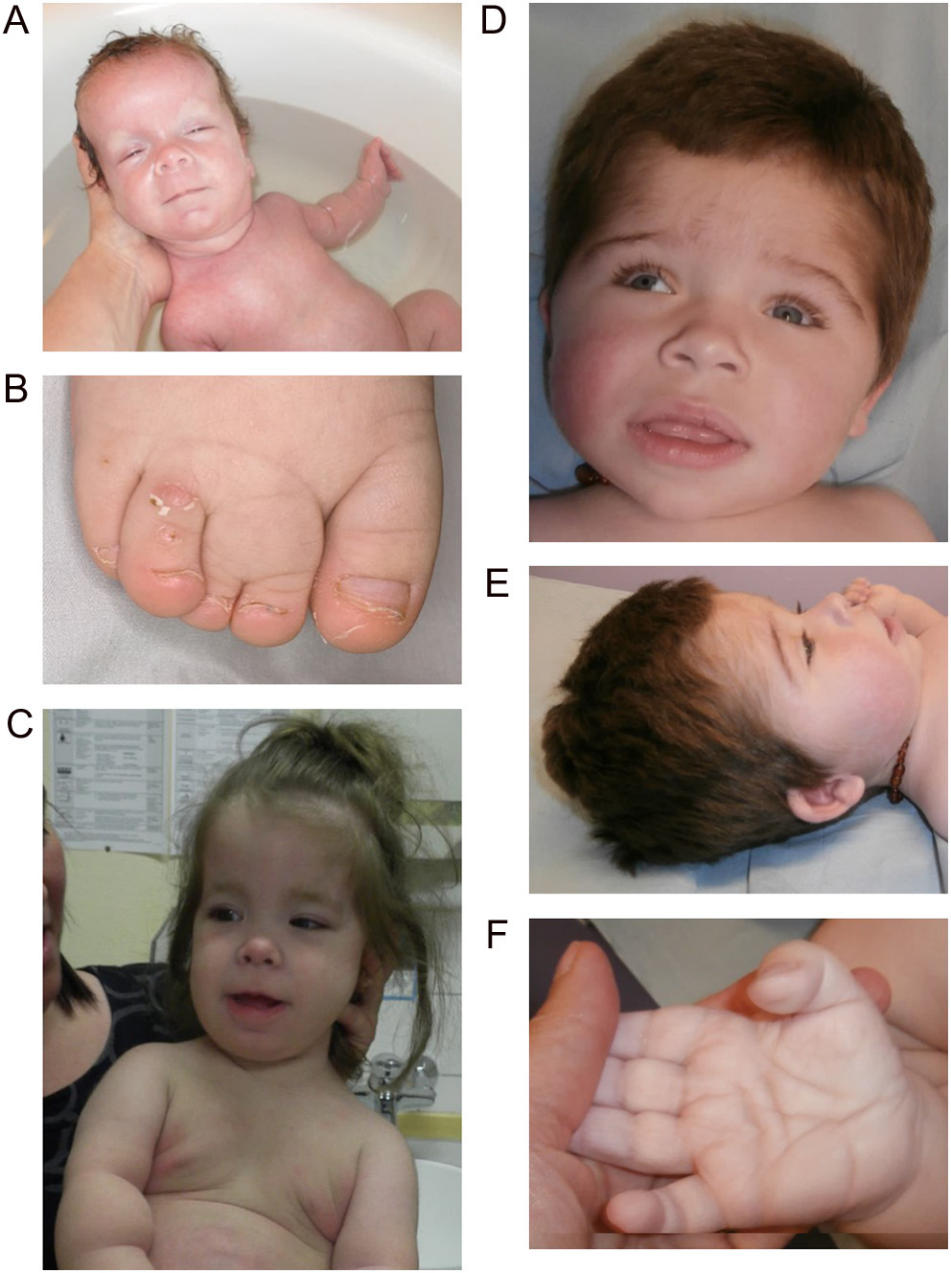
Syndromic features of patients 1 and 3. (A) Image of patient 1 at one month old demonstrating macrocephaly, facial hyperaemia and mildly asymmetric overgrowth. (B) Cutaneous syndactyly between the second and third toe on the right foot of P1. (C) Image of P1 at age 27 months demonstrating macrocephaly, obesity and overgrowth. (D and E) Head and facial features of P3 at 27 months showing macrocephaly, hypertrichosis, coarse facial appearance. (F) Deep palmar creases and excess skin in P3.

Clinical features of patients 1–3 are summarised in Table 1. Patient 1 (P1) was born at 34 weeks to healthy, non-consanguineous white German parents after polyhydramnios and macrocephaly were noted *in utero*. Her birthweight was 3230 g (+2.0 s.d.), length 52 cm (+1.5 s.d.) and head circumference 37.5 cm (+2.3 s.d.). Generalized muscle hypotonia, hyperaemia of the face, a dorsal haemangioma and dysmorphic features including diastasis recti, syndactyly, short limbs and a ‘chubby’ appearance were noted at birth (Fig. 2A and B).

**Table 1:** Summary of clinical syndromic features of patients 1–3.

	**Patient 1**	**Patient 2**	**Patient 3**
Age (years)	5	2	2
Sex	Female	Male	Male
Birth weight (kg)	3.23 (+2.0 s.d.)	4.340 (+6.8 s.d.)	3.98 (+0.33 s.d.)
Birth length (cm)	52 (+1.5 s.d.)	53 (+1.66 s.d.)	N/A
Head circumference (cm)	37.5 (+2.3 s.d.)	41 (+5.14 s.d.)	N/A
CNS features	Megalencephaly	Megalencephaly	Megalencephaly
	Arnold–Chiari malformation	Arnold–Chiari malformation	Polymicrogyria
		Polymicrogyria	
	Hydrocephalus	Hydrocephalus	Hydrocephalus
Seizures	Yes	Yes	Yes
Poly-/syndactyly	Yes	Yes	No
Vascular anomalies	Hyperaemia of face	Cutis marmorata Lower lip angioma	No
	Dorsal haemangioma	Head and neck lymphatic malformations	
Developmental delay	Yes, severe	Yes	Yes, severe
Hypotonia	Yes	Yes	Yes
Additional features	Diastasis recti	Rhizomelia	Coarse facial appearance
	Recurrent infections	Laryngomalacia	
		Gastroesophageal reflux	

Recurrent hypoglycaemia was observed on day 1. On repeated evaluations, suppressed ketones, undetectable insulin and low free fatty acids were found concomitantly with blood glucose below 2.5 mmol/L. Growth hormone and cortisol rose in response to hypoglycaemia, while plasma amino acids, urinary organic acids and acylcarnitine profile were normal. There was neither clinical nor biochemical evidence of glycogen storage diseases. Intravenous glucose requirement to maintain euglycaemia in the neonatal period was consistently below 8 mg/kg/min. Fasting tolerance in infancy peaked at 3 h before hypoglycaemia occurred, and management was based on frequent starch-enriched meals. Within the first months of life, poor oral intake led to placement of a percutaneous gastric (PEG) tube.

Diazoxide at up to 7.5 mg/kg/day for 2 weeks at the age of 1 year produced no benefit. Octreotide was tried once (5 µg/kg s.c.), and increased blood glucose from 1.8 to 5.0 mmol/L within 30 min, but was discontinued due to flushing. Glucagon (1 mg s.c.) elicited a modest glucose response during hypoglycaemia (1.9–2.2 mmol/L within 30 min); however, this was at a time of hepatopathy, evidenced by elevated liver transaminases (ALT 99 U/L, AST 183 U/L) and γGT (762 U/L). Liver biopsy, described in detail in Supplementary Online Material, did not reveal any evidence of steatosis or glycogen storage disease, but showed evidence of ductular proliferation without cholestasis or primary duct injury, reminiscent of the response to acute hepatocyte injury (Supplementary Fig. 1). 20 mg/m^2^/day methylprednisolone followed by 23 mg/m^2^/day hydrocortisone slightly improved blood glucose but rapidly increased body weight and reduced growth velocity, leading to cessation after 3 months. Subsequent management was with starch-enriched feeds during the day and PEG feeding with maltodextrin overnight. During nocturnal enteral feeding, glucose requirements were around 2–4 mg/kg/min, between 1 and 5 years of age, and euglycemic clamp studies demonstrated a requirement for 2.4 mg glucose/kg/min to maintain blood glucose between 4.0 and 4.7 mmol/L at the age of 4 years. At 4 years old, she still required continuous overnight feeding, and well-documented episodes of profound nocturnal hypoglycaemia occurred following accidental PEG displacement.

Body length was maintained around −3 s.d. throughout childhood except during glucocorticoid treatment. Body weight was between 0 and +1.3 s.d., with a weight:length ratio above the 97th percentile. Head circumference was between +3.0 and +4.0 s.d. (Fig. 2C). IGF1 and IGFBP3 were low on repeated evaluations, but growth hormone (GH) response to arginine stimulation testing at the age of 15 months was normal (13.8 μg/L within 30 min). An IGF1 generation test (GH 33 µg/kg s.c. daily for 7 days) only increased IGF1 from 25 to 56 μg/L. Decreased TSH (0.84 mU/L, reference: 0.6–5.5 mU/L) and free T4 (15.7 pmol/L, reference 10–20 pmol/L) were observed at 10 months. 1.8 µg/kg/day levothyroxine normalised fT4, with TSH remaining low.

Additional problems included severe developmental delay and an obstructing Arnold–Chiari malformation requiring ventriculoperitoneal shunting at 5 months. Recurrent airway infections required intensive respiratory support/artificial ventilation; however, no formal assessment for immunodeficiency was undertaken. At the age of 3.7 years, focal seizures independent from hypoglycaemia developed. Levetiracetam was commenced and titrated to 50 mg/kg/day at 5 years, when the patient died following cardiorespiratory arrest during an upper airway infection. There was no evidence of hypoglycaemia or Arnold–Chiari decompensation. The cause of death remains unknown.

Patient 2 (P2) was born to non-consanguineous white Spanish parents at term after polyhydramnios, and macrocephaly were identified *in utero*. His birthweight was 4340 g (+6.8 s.d.), length 53 cm (+1.66 s.d.) and head circumference 41 cm (+5.14 s.d.). Cutis marmorata, lower lip hemangioma, cutaneous lymphatic malformations and syndactyly in hands and feet were noted at birth. Imaging revealed polymicrogyria, hydrocephalus and an Arnold–Chiari malformation requiring ventriculoperitoneal shunting at 12 months. Motor development was delayed with marked muscle hypotonia at 10 months. Generalised seizures developed at 1 year, treated with sodium valproate. Liver ultrasonography at 14 months was normal.

Hypoglycaemia was first noted at 4 days and later shown repeatedly to be hypoketotic and hypoinsulinaemic. Free fatty acid and acylcarnitine profiling were normal, and glucagon stimulation demonstrated preserved, mobilisable glycogen stores (Table 2). A reduced GH response to hypoglycaemia was demonstrated at one-month old, accompanied by reduced IGF1 (<25 ng/mL) and IGFBP3 (10th percentile) concentrations. Diazoxide had no beneficial effect, and management was with regular starch-enriched meals and overnight percutaneous feeding. The glucose infusion rate used to maintain euglycaemia was 13 mg/kg/min, but no formal determination of the minimum requirement was undertaken. By 10 months, obesity had developed (weight 11.3 kg (>2.5 s.d.)). At 12 months, percutaneous feeding could be stopped; however, regular carbohydrate-enriched meals continue to be required. Although persistently low plasma IGF1 and IGFBP3 growth velocity were preserved, height remained between the 50th and 75th percentiles at 2.8 years (target height 20th percentile).

**Table 2:** Representative biochemical profiles of patients 1–3.

	**Patient 1**	**Patient 2**	**Patient 3**	**Reference range**
Age	9 months	4 days	15 months	
Glucose (mmol/L)*	2.5	2.7	1.9	3.9–5.5
Insulin (pmol/L)*	Not detectible	Not detectible	Not detectible	<14
C-peptide (nmol/L)*	0.05	0.2	0.227	<0.166**
Urine ketones*	Negative	Negative	Negative	NA
β-Hydroxybutyrate (mmol/L)*	0.024	ND	0.03	>1.8**
Free fatty acids (μmol/L)*	138	‘Not elevated’	153	<720**
Cortisol (nmol/L)*	993	464	323	>497
Growth hormone (μg/L)*	7.5	4.7	8.3	>5
Branched chain amino acids	Normal	Normal	Normal	N/A
Glucagon stimulation test^$^	Normal	Normal	Normal	See legend§
Glucose infusion rate to maintain euglycaemia (mg/kg/min)	2.4	14^$^	13^$^	>8 in CHI in infancy
Triglyceride (mmol/L)	1.1	1.1	1.0	<1.7
IGF1 (ng/mL)	<25	<25	<25	F: 36–170
				M: 27–113
IGFBP3 (µg/mL)	0.9	0.94	0.93	0.8–1.9

Values marked with an asterisk (*) were determined during hypoglycaemia, **cut-offs for differentiation between hypoketotic and physiologic/ketotic response to hypoglycaemia. ^§^A normal response to glucagon was defined as a rise in plasma glucose rose by at least 1.7 mmol/L following an intramuscular injection of at least 20 μg/kg glucagon. ^$^Glucose infusion rates were not titrated to the minimum requirement.

CHI, congenital hyperinsulinism; ND, not determined.

Patient 3 (P3) was born to non-consanguineous English parents at 42 weeks gestation following pre-natal diagnosis of hydrocephalus and macrocephaly. His birthweight was 3980 g (+0.33 s.d.). On the first day of life, he had a generalized seizure with undetectable plasma glucose. Because of recurring hypoglycaemia in the first week of life, 13 mg/kg/min intravenous glucose was used to maintain euglycaemia without titration to the lowest dose required. Repeated evaluations over ensuing months showed fasting hypoglycaemia with suppressed ketones, undetectable insulin and low but detectable C peptide levels (Table 2). Short synacthen testing, thyroid function and a genetic screen for congenital hyperinsulinism were normal or negative. Diazoxide was trialled with no discernible benefit, and 4-hourly carbohydrate-enriched feeds were required to maintain euglycaemia.

At 3 months, macrocephaly was noted (head circumference 47 cm (+5.49 s.d.)) with a coarse facial appearance, hypertrichosis and broad digits with soft palms and soles with deep creases and excess skin (Fig. 2D, E and F). Imaging showed hydrocephalus and bilateral presylvian polymicrogyria requiring insertion of a ventriculoperitoneal shunt. A clinical diagnosis of MPPH (OMIM #603387 & #615937) was made. At 2.3 years, weight was 14.7 kg (+1.23 s.d.), height 95.2 cm (+1.72 s.d.) and head circumference 53.6 cm (+3.62 s.d.). Partial seizures are well controlled on phenobarbital, but he is severely intellectually delayed with no speech and inability to sit unaided.

### Genetic diagnosis

**Figure 3:**
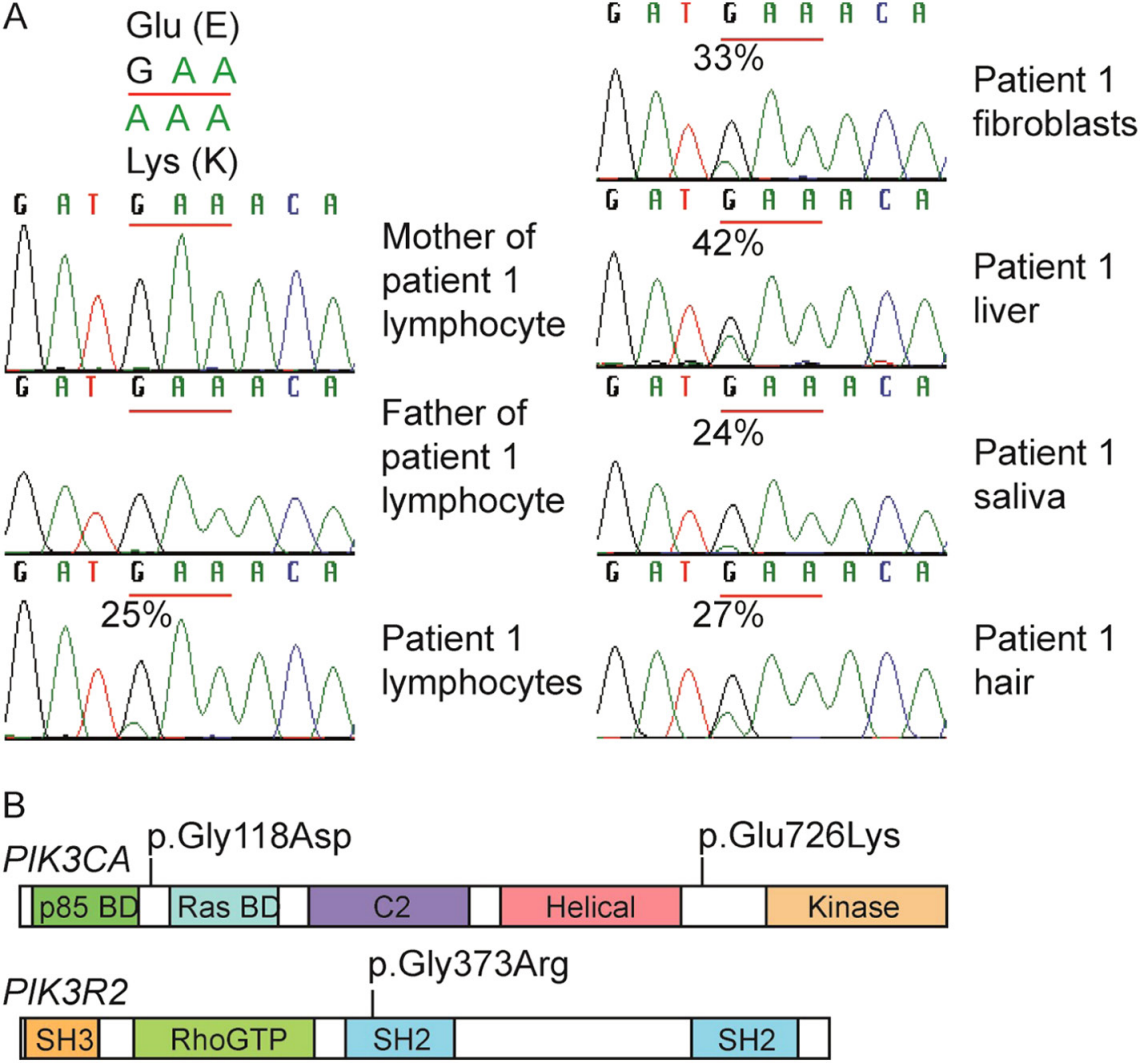
Mutations in PI3K-associated genes in patients 1–3. (A) Sanger sequencing showing *de novo PIK3CA* c.2176G > A mutation in patient 1 at 24–42% in all tissues available for DNA testing. (B) Schematic showing locations of the three PI3 kinase mutations associated with hypoglycaemia. p85 BD, p85 binding domain; RBD, Ras binding domain; SH2/3, SRC homology 2/3; BH, breakpoint cluster homologue; P, proline rich domain; PH, Phox homology domain.

*AKT2* was wild type in lymphocyte DNA from P1, so the patient and parents underwent whole exome sequencing. Five non-synonymous candidate pathogenic variants were identified with a frequency <0.01 in control databases and a posterior probability of *de novo* inheritance >0.8 (Supplementary Table 1) including a single nucleotide substitution (c.2176G > A; p.Glu726Lys) in *PIK3CA* (NM_006218.3), encoding the p110α catalytic subunit of PI3K. This variant was called in 26 of 103 reads, suggesting somatic mosaicism. Sanger sequencing identified the mutation in multiple tissues (Fig. 3A), while restriction fragment length polymorphism assay showed mutation burdens from 24% (saliva) to 42% (liver). Dermal fibroblast DNA from P2 was screened for mutations in *PIK3CA* using Sanger sequencing, revealing the *PIK3CA* c.365G > A mutation (p.Gly118Asp). This was absent from lymphocyte DNA. Both *PIK3CA* mutations have previously been described (OMIM #602501) (16, 24). DNA from a buccal swab of patient P3 was screened for overgrowth-associated mutations using an Illumina MiSeq Panel (Supplementary Online Materials Table 2). A heterozygous c.1117G > A (p.Gly373Arg) mutation was identified in *PIK3R2* (NM_005027.3), encoding the p85β regulatory subunit of class 1A PI3K. p.Gly373Arg is the most common mutation in *PIK3R2* associated with MPPH type 1 (OMIM #603387) (16). The mutation was identified at a mutation burden of 50%, and this was confirmed independently in a diagnostic laboratory in lymphocyte DNA, also at 50%, consistent with a constitutional mutation. No parental DNA was available for testing. The locations of all 3 mutations in their respective proteins are schematised in Fig. 3B.

### Cellular studies

**Figure 4:**
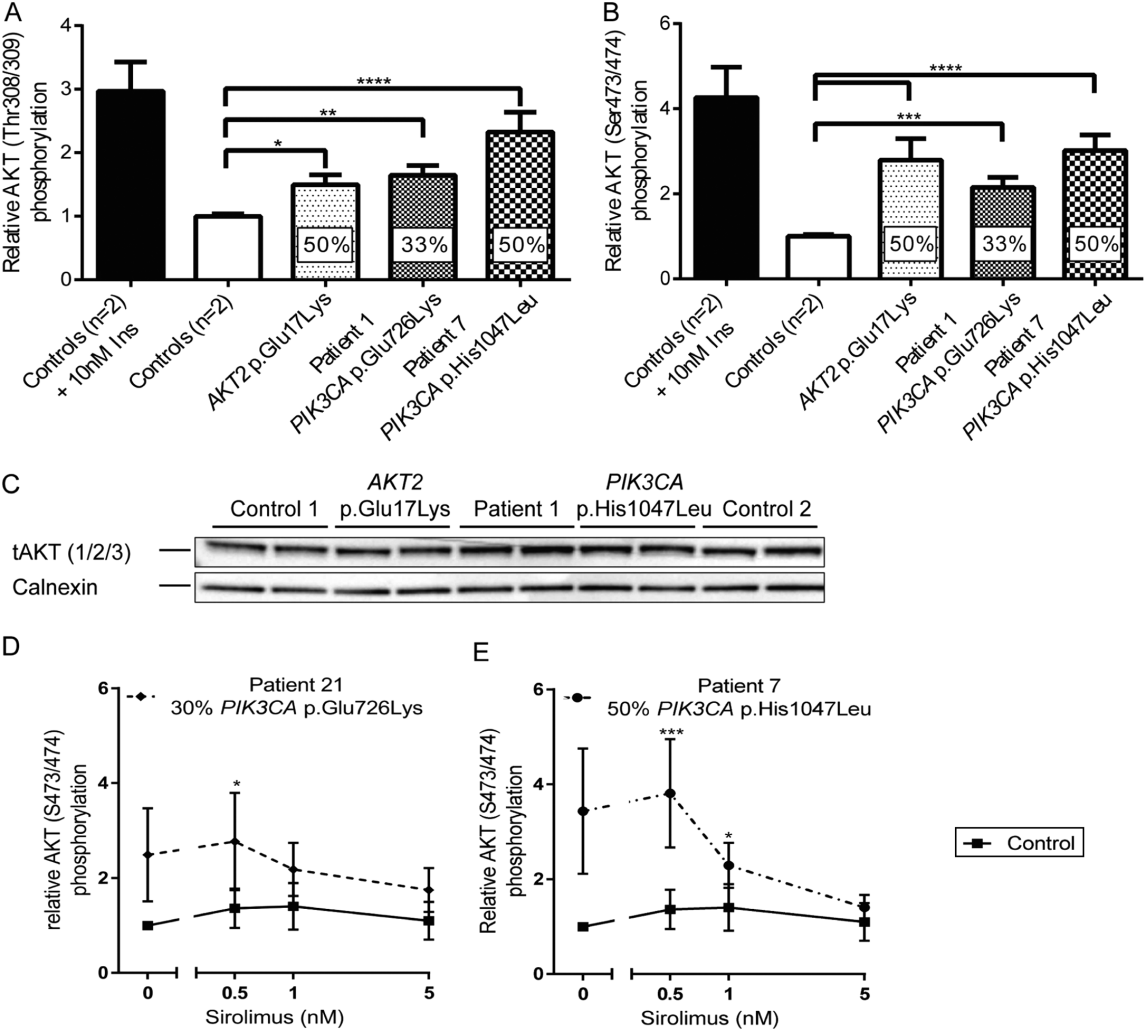
Signalling downstream from phosphatidylinositol-3-kinase in dermal fibroblasts from patient 1. AKT phosphorylation at Thr308/309 (A) or Ser473/474 (B) was determined by ELISA. Percentages in columns indicate the mutation burden in the cells. Data represent pooled, normalised results from three independent experiments plotted as mean ± s.e.m. One-way ANOVA and *post hoc* Dunnett’s test were used to assess significance. Insulin stimulation is shown for illustration only and was excluded from this statistical analysis. (C) Immunoblotting for total AKT (1/2/3) from samples used for ELISA (A and B). Representative blot from one of three independent replicates. (D and E) AKT Ser473/474 phosphorylation following 72 h of Sirolimus treatment was quantified using ELISA and normalised to AKT expression determined by immunoblotting. (D) dermal fibroblasts from P21; (E) dermal fibroblasts from P7. Results represent mean ± s.e.m. of three independent replicates. DMSO on its own did not result in a change in phosphorylation (data not shown). Statistical analysis was performed as one experiment and separate graphs are plotted for clarity only. A two-way ANOVA was performed and followed by a *post hoc* Dunnett’s test comparing all patients to the same control cell line. *P < 0.05, **P < 0.01, ***P < 0.001, ****P < 0.0001.

Dermal fibroblasts of P1 were cultured and shown to harbour a mutation burden of 33%, consistent with 66% of the cells carrying a heterozygous mutation at passage 4 (Fig. 3A). They showed a small, significant increase in basal phosphorylation of AKT at Threonine 308/309 and Serine 473/474, as in cells expressing *AKT2* p.Glu17Lys or the overgrowth-related *PIK3CA* p.His1047Leu (Fig. 4A, B and C). By passage 6, this was no longer seen in cells from P1, likely due to progressive decline in mutation burden. Seventy-two hours exposure to Sirolimus, a dual mTORC1/mTORC2 inhibitor, did suppress basal hyperphosphorylation of AKT at Serine 473/474 (the target site of the upstream kinase mTORC2) in early passage cells from another patient carrying the same *PIK3CA* p.Glu726Lys mutation (P21) (Fig. 4D), however, or cells expressing *PIK3CA* p.His1047Leu (Fig. 4E).

### Metabolic assessment of a wider PI3K-related overgrowth spectrum (PROS) cohort

To profile the wider metabolic consequences of mosaic activation of PI3K, a further 19 patients with mosaic activating *PIK3CA* mutations were assessed, as summarised in Table 3. All patients had clinical disorders encompassed by the *PIK3CA*-Related Overgrowth Spectrum (PROS) (15), and were subdivided into those with severe, localized overgrowth and those with more diffuse overgrowth including the brain, similar to patients 1–3. One further patient in this group had the same p.Glu726Lys variant as P1 and one had the p.Gly118Asp variant seen in P2.

**Table 3:** Genetic diagnosis and biochemical profile following an overnight fast in patients with PROS.

ID	Sex	Age (years)	**Syndrome**	**Site of overgrowth**	**B.M.I.** (kg/m^2^) or *Z-score	**Fat mass** (%)	PIK3CA mutation	Glucose (mmol/L)	Insulin (pmol/L)	**HbA1c** (mmol/mol)	**Adiponectin** (mg/L)	Leptin (μg/L)	IGF1 (ng/mL)
NR								3.9–5.5	0–60	30–48	See legend	See legend	See legend
P4	M	48	CLOVES	L foot	26.7	13	C420R	4	11	40	23.2	ND	76
P5	M	29	CLOVES	L leg and trunk	25.7	23	E545K	4.5	27	34	3.8	5.7	163
P6	M	15	CLOVES	R thorax	−1.6*	19	E542K	6.2	165	ND	17.3	1.8	119
P7	F	37	FAH	Both legs	55	54	H1047L	3.9	13	36	16.9	166	141
P8	F	31	FAH	R arm	32.5	46	H1047L	4.7	ND	ND	ND	ND	221
P9	F	34	FAH	L leg	32.7	47	H1047R	4.7	217	43	1.5	45.5	141
P10	F	9	FAH	L leg	>+3*	29	H1047R	4.8	42	36	ND	ND	122
P11	F	22	MO	Both arms	21.9	32	H1047R	4.7	31	ND	6.3	6.6	242
P12	M	34	KTS	L leg	27.8	38	V346insK	4.3	29	38	ND	16	168
P13	F	40	MD	L hand	32.8	57	H1047R	4.5	73	37	ND	ND	ND
P14	F	39	MD	2 R fingers	25	40	M1043_N1044delinsIY	4.7	41	33	7.4	13.5	176
P15	F	35	MCAP	Diffuse	34.9	38	G118D	5.1	31	40	5.1	33.3	145
P16	M	29	MCAP	Diffuse	27.1	23	E81K	5.2	31	38	3.9	4.1	158
P17	F	18	MCAP	Diffuse	23.6	43	E81K	5.1	80	35	8.6	31.4	382
P18	F	21	MCAP	Diffuse	43	52	G914R	5.4	51	36	5.1	ND	85
P19	M	1.5	MCAP	Diffuse	ND	ND	R88Q	5.1	<3	48	ND	ND	<25
P20	M	21	MCAP	Diffuse	23.9	44	E726K	3.9	7	ND	6.6	2	28
P21	M	3	MCAP	Diffuse	+1*	43	R93Q	4.3	18	ND	ND	ND	60
P22	M	12	MCAP	Diffuse	ND	ND	D350G	3.9	<14	ND	ND	ND	80

BMI-specific reference ranges for adiponectin in females—<25 kg/m^2^: 4.4–17.7 mg/mL; 30–35 kg/m^2^: 2.6–14.9 mg/mL; >35 kg/m^2^: 2.6–17.1 mg/mL. Adiponectin reference in males—<25 kg/m^2^: 2.6–12.6 mg/mL; 25–30 kg/m^2^: 2.4–10.6 mg/mL. BMI-specific reference ranges for leptin in females—<25 kg/m^2^: 2.4–24.4 ng/mL; 25–30 kg/m^2^: 8.6–38.9 ng/mL; >35 kg/m^2^: 22.7–113.6 ng/mL. Leptin reference in males—<25 kg/m^2^: 0.4–8.3 ng/mL; 25–30 kg/m^2^: 1.5–13.0 ng/mL. Age-specific IGF-1 references ranges in females—9–11 years: 87–396 ng/mL; 16–20 years: 266–467 ng/mL; 21–24 years: 148–330 ng/mL; 25–40 years: 123–304 ng/mL. IGF-1 reference ranges in males—2–60 months: 27–113 ng/mL; 12–15 years: 115–495 ng/mL; 21–24 years: 186–397 ng/mL; 25–40 years: 124–300 ng/mL; 41–50 years: 89–239 ng/mL.

CLOVES, congenital lipomatous overgrowth with vascular, epidermal and skeletal anomalies; FAH, fibroadipose hyperplasia; KTS, Klippel–Trenaunay syndrome; MCAP, macrocephaly and capillary malformation syndrome; MD, macrodactyly; MO, muscle overgrowth; ND, not determined; NR, normal range.

None of the PROS patients had fasting hypoglycaemia; however, three patients with MCAP had low or undetectable plasma insulin concentrations with normal or low-normal fasting glucose (P19, P20, P22; Table 3). One of these patients P19 previously had hypoglycaemia during an episode of diarrhoea and vomiting. Five of the eight patients with MCAP, including all three with low fasting insulin levels, had low IGF1 concentrations. Two patients with more extensive overgrowth (P6 and P9) had hyperinsulinemia consistent with insulin resistance.

Oral glucose tolerance tests (OGTT) were completed for thirteen patients, five with MCAP. As shown in Supplementary Fig. 2 and Supplementary Table 3, glucose and insulin were within normal ranges. Finally, given the large pathological expansion of specific adipose depots in many patients with focal severe PROS, and the interest in distinct physiological properties and secreted adipokine profiles of these depots, adipokine levels relative to whole body fat mass were compared between adults in the PROS group and a large control dataset (Supplementary Fig. 3). However, adipokine levels were similar to those in the control population in the large majority of PROS patients including those with extreme focal fat mass (P7, P8), arguing that the adipokine profiles are not distinct in different forms of PROS. Two exceptions were P4, a patient with CLOVES who had a disproportionately high adiponectin concentration, and P20, a patient with CLOVES who had a disproportionately low leptin concentration.

## Discussion

‘PI3K’ here denotes a group of enzymes composed of one of three catalytic subunits (p110α, β or δ) and one of five regulatory subunits (p85α, p55α, p50α, p85β or p55γ) (25). PI3K generates PIP_3_ in response to insulin receptor activation, leading in turn to AKT/PKB activation. Genetic and pharmacological evidence has established that the catalytic p110α subunit, encoded by *PIK3CA*, is critical for the glucose-lowering by insulin, while AKT2 is the ‘metabolic AKT isoform’ (26). The only example to date of hypoketotic hypoglycaemia due to a mutation activating insulin signalling is a *de novo* activating mutation in *AKT2* (27) that has been described in five patients. Activation of other components of this pathway, such as PI3K, would be anticipated to cause a similar metabolic profile; however, given the pleiotropic growth-promoting actions of PI3K, the associated syndrome would be expected to be more complex. Mosaic genetic activation of PI3K has recently been described in a wide range of segmental overgrowth disorders with overlapping features (13, 28). Seven patients with *PIK3CA* mutations, overgrowth and infantile hypoglycaemia were described while this work was underway (18), but no further metabolic characterisation was undertaken.

We describe three patients with megalencephalic PI3K-related overgrowth who had severe, likely non-insulin-dependent hypoketotic hypoglycaemia, similar to that caused by activating *AKT2* mutations, persisting to at least 2 years old in all cases. This affected only a subset of patients with PI3K-related overgrowth, likely due to the predominantly mosaic nature of the condition. This mosaicism, with only some cells and tissues affected, complicates efforts to distinguish among several possible mechanisms underlying hypoglycaemia. The simplest explanation is that tonic activity of hepatic PI3K leads to inappropriate suppression of hepatic glycogenolysis and gluconeogenesis in the postabsorptive state. Most of the liver would have to be affected to exert a significant effect. A liver biopsy from P1 presented the opportunity to examine liver in one patient with *PIK3CA*-related hypoglycaemia. It showed a mutation burden of 42%, corresponding to 84% of cells bearing a heterozygous mutation; however, neither glycogenosis nor steatosis were seen, while prominent ductular reaction without cholestasis or primary duct injury was reminiscent of the response to severe and acute hepatocyte injury (29). Without knowing which cells harboured the *PIK3CA* mutation, no more precise correlation was possible between the abnormal histology and the mutation. If liver-autonomous failure to derepress glycogenolysis/gluconeogenesis were the major abnormality explaining PROS-related hypoglycaemia, then only a small glucose requirement to maintain euglycaemia would be expected, as reported for patients with activating *AKT2* mutations. (27) This was confirmed for P1 in this report; however, infusion rates in P2 and P3 were reported to be high. Whether glucose infusions were titrated to the lowest possible rate to maintain euglycaemia was unclear, however.

PI3K-related hypoglycaemia may alternatively reflect suppression of adipose lipolysis, which provides energy and gluconeogenic substrates to fasting hepatocytes (30, 31). This was shown by the lack of an adequate rise of free fatty acids in P1 during hypoglycaemia (Table 2). Fasting free fatty acid levels in the PROS cohort were not studied; however, in two patients with genetic AKT2 activation, we observed correlation of free fatty acid suppression with fasting hypoglycaemia (32). Although increased PI3K-mediated, GLUT4-dependent glucose uptake into skeletal muscle and adipose tissue could also cause hypoglycaemia, patients with severe local adipose or muscle overgrowth due to strongly activating *PIK3CA* mutations, but with no liver involvement, exhibited neither spontaneous hypoglycaemia nor hypoinsulinaemia. This argues that regional adipose tissue or muscle PI3K-mediated glucose uptake either does not occur, or is insufficient to produce hypoglycaemia.

Finally, aberrant activity of PI3K in the brain may also play a role: low IGF1 levels in 63% of the MCAP patients suggest dysfunctional GH secretion or action. This may reflect tonically active PI3K in the hypothalamus and/or the pituitary, mimicking IGF1 action and erroneously activating negative feedback inhibition of GH-releasing hormone or GH release. In contrast, stimulation of GH secretion on provocative testing may yield normal results, as shown in P1, suggesting that suppressed basal secretion may be overridden. The central hypothyroidism in P2, and in three previously described MCAP patients (18), and the modest GH and cortisol responses to hypoglycaemia suggest that hypothalamic–pituitary function may show wider perturbation. Attenuated neuroendocrine counterregulatory responses to hypoglycaemia are a plausible explanation for the GH and cortisol responses; however, this cannot be proven, and studies of further affected patients will be required to elucidate whether abnormality of regulation, a subtle developmental disorder of the hypothalamus/pituitary or both are seen in MCAP caused by PI3K activation. Evidence that different adipose depots show distinct profiles of adipokine secretion (33, 34) raised the possibility that pathological expansion of only some adipose depots (e.g. legs vs upper body) would correspond to a distinct pattern of circulating adipokines. In general, we found this not to be the case, with the large majority of patients showing a similar relationship between adipokine concentrations and whole body adipose tissue mass to that seen in the general population. Two outliers were found with perturbed adiponectin or leptin levels; however, the explanation was not immediately clear, and wider investigation may be warranted.

Importantly, our findings suggest that mTOR inhibitors such as Sirolimus may have therapeutic benefit instead of, or in addition to, nutritional therapies including percutaneous overnight feeding. Although Sirolimus is primarily an mTORC1 inhibitor, prolonged use sequesters components of the mTORC2 complex (35), which acts upstream from AKT in insulin signalling. Our findings suggest that Sirolimus at very low doses (1.1–2.2 ng/mL) may be sufficient, unlike the high doses used in hyperinsulinism (5–15 ng/mL) (36). In future, low doses of more specific class 1A PI3K inhibitors, many examples of which are in clinical trials for cancer, may offer a more specific targeted therapy.

In conclusion, we report that a subset of patients with diffuse, megalencephalic forms of *PIK3CA*-related segmental overgrowth and MPPH exhibit severe insulin-independent hypoketotic hypoglycaemia. This metabolic phenocopy of hypoglycaemia driven by genetic AKT2 activation is not seen in patients with extreme regional adipose or muscular overgrowth associated with ‘hot spot’ activating PIK3CA mutations, suggesting that diffuse mutation carriage is more important than the degree of PI3K activation in determining the phenotype. We suggest that endocrinologists should be alert to the possibility of syndromic PI3K-related overgrowth when evaluating infants with hypoinsulinaemic hypoketotic hypoglycaemia, or with equivocal biochemical testing for congenital hyperinsulinism, and that they should understand the challenges of testing for a mosaic genetic disorder. Prior knowledge and our cellular studies suggest that mTOR inhibitors may offer therapeutic benefit, and are worthy of clinical study.

## Supplementary Material

Supplementary Online Material

Supplementary figure

Supporting Table 1

Supporting Table 2

Supporting Table 3

## Declaration of interest

I B would like to disclose ownership of stock in GlaxoSmithKline, and Incyte and R K S would like to disclose receipt of speaker fees from Novo Nordisk and Sandoz.

## Funding

This work was supported by the Wellcome Trust (grant number Wbib98498); the Medical Research Council (MRC_MC_UU_12012/5); the United Kingdom National Institute for Health Research (NIHR) Cambridge Biomedical Research Centre; the Rosetrees Trust (M223); and the EU/EFPIA Innovative Medicines Initiative Joint Undertaking (EMIF grant no 115372).
