# Supplementary material for: Hypoinsulinaemic, hypoketotic hypoglycaemia due to mosaic genetic activation of PI3-kinase
Source: Eur J Endocrinol. 2017 May 30;177(2):175–86. doi: 10.1530/EJE-17-0132 (PMC5488397; doi:10.1530/EJE-17-0132)
Supplement: Supplementary Online Material [file eje-177-175-s001.pdf]

Hypoinsulinaemic, hypoketotic hypoglycaemia due to mosaic genetic activation of Type 1A PI3-Kinase

Leiter *et. al.*

Supplementary Material

Supplementary Methods

Clinical studies

Age- and BMI-matched reference ranges for adiponectin and leptin were derived from the MRC Ely study population (25). Age-specific IGF1 reference ranges were derived from the DiaSorin LIASON IGF1 assay protocol (#313231) up to 24 years and from published ranges above 25 years (26). Z-scores for growth indices were determined using World Health Organisation tables (http://www.who.int/childgrowth/standards/weight_for_age/en/). Oral glucose tolerance tests (OGTTs) were performed following an overnight fast; patients received 1.75 g glucose/kg body weight (max 75g) and plasma glucose and insulin samples were taken at the indicated time-points. Body composition was determined using dual energy X-ray absorptiometry (DEXA) at the Wellcome Trust Clinical research facility in Cambridge. Adipokine levels represented against DEXA-derived fat mass were obtained from the MRC Fenland Study (http://www.mrc-epid.cam.ac.uk/research/studies/fenland/, manuscript in preparation).

Genetic Studies

For exome-wide sequencing of P1, DNA extracted from peripheral blood leukocytes was sequenced and mapped to the human genome reference sequence (GRCh37). Variants were called using the Genome Analysis Toolkit HaplotypeCaller (v3.3-0-537228af) (28) and annotated with Ensembl Variant Effect Predictor (Ensembl release 75) (29) and the NCBI dbSNP database build 138 (ftp://ftp.ncbi.nlm.nih.gov/snp/organisms/human_9606/). Sample and variant quality control was performed as described (19). Raw exome sequence is available from the European Genome-Phenome Archive (https://www.ebi.ac.uk/ega/home; Study ID: EGAS00001000488). DeNovoGear (v0.5.4) (30) was used to identify likely *de novo* variants (cutoff set at 0.8). Putative compound heterozygous mutations were also extracted. Allele frequencies were assessed in 1000 Genomes Phase 1 integrated callset v3 (31), the Exome Variant Server, NHLBI GO Exome Sequencing Project (ESP), Seattle, WA (URL: http://evs.gs.washington.edu/EVS/) [April 2013], the UK10K Cohorts [REL-2012-06-02] and Exome [REL-2013-04-20] groups (19), and exome sequencing data from 409 control individuals from the CoLaus Cohort (32). Variants were prioritised as described previously (33).

For determination of mutation burden of the *PIK3CA* p.Glu726Lys mutation DNA was amplified with GoTaq Green (Promega) using the following primers: 5’-ATAGGCAAGTCGAGGCAATG-3’ and 5’-[6FAM]TGCTTGATTCCAAGGACCAT-3’. The DNA fragment was digested using BseGI (FastDigest, Thermo Fisher) to cut the

wild-type template specifically resulting in a 109bp FAM-labelled fragment. Digest was mixed with Liz500/HiDi-Formamide size standard (Applied Biosystems), run on an ABI3730 capillary sequencer, and areas under the curve for uncut and cut DNA were used to calculate mutation burden in Genemapper (Applied Biosystems).

The custom panel of overgrowth-related genes sequenced in P3 is shown in Supplementary Table S2. For sequencing using the Illumina MiSeq platform, 10 ng of DNA was amplified for 18 cycles of PCR with the Ion Ampliseq Custom DNA panels, covering 195 amplicons of 60 genes. The panel was split into two primer pools and amplified with 5X Ion Ampliseq HiFi master mix, followed by FuPa treatment (Ion Ampliseq DNA Library Kit 2.0). Both primer pools for each sample were pooled and purified with 1.8X Agencourt AMPure XP magnetic beads (Beckman Coulter). Amplicons were 3’ adenylated using the NebNext Ultra II end repair/dA tailing module (NEB), followed by NextFlex DNA Barcode adapter (Bio Scientific) ligation using the NebNext Ultra II ligation module (NEB). Ligation products were then purified and size selected using firstly 0.8X Agencourt AMPure XP beads.

Library concentration was determined with Kapa Biosystems Library qPCR quantification kit on the Lightcycler 480 real-time PCR system (Roche). Libraries were subsequently diluted to 2 nM and pooled in equimolar amounts. Pooled libraries were spiked with 1% PhiX DNA (Illumina), and sequenced on the MiSeq desktop sequencer using version 2 chemistry at 250 bp read length paired end. The *PIK3R2* mutation was confirmed using Sanger sequencing as above. Primer sequences are available upon request. VCF files tailored for mosaic variant calling were created in MiSeq Reporter (Illumina) and annotated in Illumina Variant Studio version 2.2, resulting in a list of 300-1000 variants per sample. An R script was then used to filter variants according to the following parameters: read depth > 5; quality score > 10; absence of strand bias (as determined by MiSeq Reporter), cross-sample subtraction of artefactual variants called in > 4 samples per batch of 24; exonic non-synonymous variants only. This resulted in a list of 1-4 candidate variants, the clinical relevance and sequencing quality of which were then accessed manually. Mosaic variants likely to be causative were confirmed alongside healthy control DNA, by Sanger sequencing.

Histology

Liver tissue was obtained from patient 1 by percutaneous biopsy and underwent routine formalin-based fixing and processing to a paraffin block. Serial sections were cut at 3 □m and stained with haematoxylin and eosin, elastic picrosirius red, or Shikata’s orcein. Immunostaining using Bond technology for cytokeratin 7 was also undertaken (Agilent Technologies).

Supplementary Results

P1 Histology

Histological examination showed two short cores of liver tissue with preserved architecture and portal tract fibrous expansion, with occasional porto-portal linking fibrosis (Figure S1A). A variable, generally slight portal chronic inflammatory cell infiltration was present (Figure S1B). There was marked increase in ductules within portal tracts and extending into the parenchyma (Figure S1B) although intraportal native bile ducts did not show significant irregularity or ductopenia. Ducts and ductules were uniformly positive for cytokeratin 7 which also identified several intermediate cells in periportal positions (Figure S1C). There was no cholestasis, steatosis or inflammation within the parenchyma, although occasional pseudoglandular rosettes were evident (Figure S1D&E). Hepatocytes did not appear markedly enlarged and there was no clearing of the cytoplasm or thickening of the cytoplasmic membrane. No intracytoplasmic inclusions nor multinucleation was evident. No abnormal Kupffer cells or storage cells were apparent. Very focal, slight deposition of copper associated protein was present in a few periportal hepatocytes (not shown). All liver function tests normalized completely within the second year of life.

Figure legends

Supplementary Figure S1 – P1 liver histology at 13 months old A) picrosinus red stain demonstrating portal tract fibrous expansion, with occasional porto-portal linking fibrosis, B) low magnification hematoxylin and eosin stain showing a chronic inflammatory cell infiltrate, C) low and D) high magnification cytokeratin 7 revealing portal proliferation and occasional pseudogladular rosettes, E) high magnification hematoxylin and eosin displaying pseudoglandular rosettes.

Supplementary Figure S2 – Oral glucose tolerance in patients with *PROS*. Comparison of average glucose and insulin responses to 1.75g/kg oral glucose over subsequent 0-120 minutes. Patients with PROS are divided into those with MCAP (A, B; usually more diffusely affected) and those with non MCAP (C, D; more severe and localised overgrowth) subtypes. Patients with macrodactyly only were excluded due to their limited disease. Box and whisker plots represent the median and interquartile ranges (box) and the minimum/maximum (whiskers). Lines represent corresponding 5th, 50th and 95th centiles in the Ely control cohort comprising a predominantly Caucasian middle-aged group in the East of England.

Supplementary Figure S3 – Plasma adipokine concentrations with reference to body fat content. DEXA-determined body fat percentage was compared against adiponectin and leptin levels separated by sex for patients with PROS and matched controls from the Fenland study.
