# Supplementary material for: Hypoinsulinaemic, hypoketotic hypoglycaemia due to mosaic genetic activation of PI3-kinase
Source: Eur J Endocrinol. 2017 May 30;177(2):175–86. doi: 10.1530/EJE-17-0132 (PMC5488397; doi:10.1530/EJE-17-0132)
Supplement: Supporting Table 1 [file eje-177-175-t001.pdf]

Supplementary Table S1 –*de novo* candidate genes in patient 1

**Gene**	**Protein**	**Posterior probability**	**Genotype (P1/mother/father)**	**Chr**	**GRCh37**
DDB2	Thr246Met	1	CT/CC/CC	11	47256342
ARHGAP12	Gly127Val	1	AC/CC/CC	10	32197404
PIK3CA	Gly726Lys	0.999992	AG/GG/GG	3	178938934
ACSM5	Ala173Val	0.999901	CT/CC/CC	16	20430652
NBPF9	Asn183Asp	0.995885	GG/GG/AA	1	144815953
