# Supplementary material for: Hypoinsulinaemic, hypoketotic hypoglycaemia due to mosaic genetic activation of PI3-kinase
Source: Eur J Endocrinol. 2017 May 30;177(2):175–86. doi: 10.1530/EJE-17-0132 (PMC5488397; doi:10.1530/EJE-17-0132)
Supplement: Supporting Table 2 [file eje-177-175-t002.pdf]

Supplementary Table S2 – Panel of Genes Sequenced in P3

AKT1	FLT3	LAMTOR2	PTEN
AKT2	GNA11	MAP2K1	PTPN11
AKT3	GNAQ	MAPKAP1	RHEB
ALK	GNAS	MET	RICTOR
BRAF	HRAS	MLST8	RPS6KB1
CCND2	IDH1	mTOR	RPS6KB2
CDK2	IDH2	NRAS	RPTOR
DEPTOR	IGF1R	PDGFRA	RRAGA
EGFR	IGF2R	PDK1	RRAGB
ERBB2	JAK2	PHLPP1	RRAGC
ERBB3	JAK3	PHLPP2	RRAGD
EZH2	KDR	PIK3CA	SMAD4
FGFR1	KIT	PIK3CB	SOS1
FGFR2	KRAS	PIK3R1	SRC
FGFR3	LAMTOR1	PIK3R2	STAT3
