# Supplementary material for: Hypoinsulinaemic, hypoketotic hypoglycaemia due to mosaic genetic activation of PI3-kinase
Source: Eur J Endocrinol. 2017 May 30;177(2):175–86. doi: 10.1530/EJE-17-0132 (PMC5488397; doi:10.1530/EJE-17-0132)
Supplement: Supporting Table 3 [file eje-177-175-t003.pdf]

Supplementary Table S3 – Results of oral glucose tolerance testing in 13 patients with *PROS*. Abbreviations used: CLOVES - Congenital lipomatous overgrowth with vascular, epidermal, and skeletal anomalies; FAH – Fibroadipose hyperplasia; KTS - Klippel-Trenaunay syndrome; MD – macrodactyly; MCAP – Macrocephaly and capillary malformation syndrome; ND – not determined

**Patient**	**Age (years)**	**Diagnosis**	**Glucose (mmol/l)**				**Insulin (pmol/l)**			
			**0'**	**30'**	**60'**	**120'**	**0'**	**30'**	**60'**	**120'**
P4	48	CLOVES	4.0	6.2	5.4	4.4	26	252	228	117
P7	37	FAH	5.2	8.8	7.9	6.5	18	85	112	90
P8	31	FAH	4.9	7.5	6.0	6.2	ND	ND	ND	ND
P9	34	FAH	4.7	6.5	9.0	6.9	217	751	1851	1797
P10	9	FAH	4.8	6.9	8.3	6.3	42	122	207	113
P12	34	KTS	4.3	6.2	5.7	5.7	29	260	244	192
P13	40	MD	4.5	6.0	6.3	6.0	63	459	428	429
P14	39	MD	4.7	8.8	10.1	8.6	41	312	201	41
P15	35	MCAP	4.9	6.6	4.1	4.9	32	30	136	76
P16	29	MCAP	4.7	7.1	5.4	4.6	41	201	162	41
P17	18	MCAP	4.2	5.8	5.8	5.8	ND	840	952	535
P18	21	MCAP	4.8	8.5	7.3	5.8	51	333	312	149
P22	12	MCAP	3.8	5.6	6.7	ND	<14	160	196	ND
